# Comparative Study on Extraction of Cellulose Fiber from Rice Straw Waste from Chemo-Mechanical and Pulping Method

**DOI:** 10.3390/polym14030387

**Published:** 2022-01-19

**Authors:** Nur Amirah Mamat Razali, Risby Mohd Sohaimi, Raja Nor Izawati Raja Othman, Norli Abdullah, Siti Zulaikha Ngah Demon, Latifah Jasmani, Wan Mohd Zain Wan Yunus, Wan Mohd Hanif Wan Ya’acob, Emee Marina Salleh, Mohd Nurazzi Norizan, Norhana Abdul Halim

**Affiliations:** 1 Center for Defence Foundation Studies, Universiti Pertahanan Nasional Malaysia, Kuala Lumpur 57000, Malaysia; nuramira.mamatrazali@gmail.com (N.A.M.R.); norli.abdullah@upnm.edu.my (N.A.); zulaikha@upnm.edu.my (S.Z.N.D.); mohd.nurazzi@gmail.com (M.N.N.); 2 Faculty of Engineering, Universiti Pertahanan Nasional Malaysia, Kuala Lumpur 57000, Malaysia; risby@upnm.edu.my (R.M.S.); izawati@upnm.edu.my (R.N.I.R.O.); 3 Forest Research Institute Malaysia (FRIM), Kuala Lumpur 57000, Malaysia; latifah@frim.gov.my; 4 Center for Tropicalisation, National Defence University of Malaysia, Kem Perdana Sungai Besi, Kuala Lumpur 57000, Malaysia; wanmdzin@upnm.edu.my; 5 Centre for Defence Research and Technology, National Defence University Malaysia, Kuala Lumpur 57000, Malaysia; wanmohdhanif@upnm.edu.my; 6 Department of Mineral and Geoscience Malaysia, Mineral Research Centre, Ipoh 30020, Malaysia; emeemarina@jmg.gov.my

**Keywords:** cellulose, chemo-mechanical, pulping, rice straw, XRD, FTIR

## Abstract

Inspired by nature, cellulose extracted from plant wastes has been explored, due to its great potential as an alternative for synthetic fiber and filler that contributes to structural performance. The drive of this study was to extract, treat, and evaluate the characteristics of rice straw (RS) (*Oryza sativa* L.) cellulose as a biodegradable reinforcement to be utilized in polymer base materials. Two routes of extraction and treatment were performed via the pulping (Route 1) and chemo-mechanical methods (Route 2), in order to discover comparative characteristics of the synthesized cellulose fiber. Comprehensive characterization of RS cellulose was carried out to determine crystallinity, surface morphology, and chemical bonding properties, using X-ray diffraction (XRD), field emission scanning electron microscopy (FESEM), and Fourier transform infra-red (FTIR), respectively. The XRD test results showed that the crystallinity index (CI) of cellulose powder (CP) decreased after the surface modification treatment, Route 2, from 64.50 to 50.10% CI for modified cellulose powder (MCP), due to the surface alteration of cellulose structure. From Route 1, the crystallinity of the fibers decreased up to 33.5% (dissolve cellulose, DC) after the pulp went through the surface modification and dissolution processes, resulting from the transformation of cellulose phase into para-crystalline structure. FESEM micrographs displayed a significant reduction of raw RS diameter from 7.78 µm to 3.34 µm (treated by Route 1) and 1.06 µm (treated by Route 2). The extracted and treated cellulose via both routes, which was considerably dominated by cellulose II because of the high percentage of alkaline used, include the dissolve cellulose (DC). The dissolution process, using NMMO solvent, was performed on the pulp fiber produced by Route 1. The fiber change from cellulose I to cellulose II after undergoes the process. Thus, the dissolution process maintains cellulose II but turned the pulp to the cellulose solution. The acquired characteristics of cellulose from RS waste, extracted by the employed methods, have a considerably greater potential for further application in numerous industries. It was concluded that the great achievement of extracted RS is obtained the nanosized fibers after surface modification treatment, which is very useful for filler in structural composite applications.

## 1. Introduction

Being environmentally friendly, applications of biodegradable cellulose extracted from natural fiber offer new technological and commercial prospects for various areas, including the military, aerospace, automotive, electronics, and packaging industries [1,2,3]. Cellulose can also be used to make biocomposites. This is important, from an ecological point of view. Currently, many materials are being tested, with the addition of various celluloses. It has been shown that the fibers obtained from these alternative sources have properties similar to, or better than, the properties of cotton and linen. The natural fibers from native sources are sustainable materials, which are easily available in nature and have advantages, such as being lightweight and renewable, as well as having high specific properties [4]. In addition to fibrous cellulose, good effects are also achieved by the use of microcrystalline cellulose, nanocellulose, etc. These are developmental materials; therefore, it is worth emphasizing the importance of cellulose as an additive [5,6,7]. Typically, the synthetic fibers have an undesirable impact on the environment, human health, and the intensification of the global energy crisis [8,9]. Synthetic fibers require high energy in the production state that will produce high level of greenhouse gases (GHG) emission, which will cause the trapping of heat and contribute to respiratory disease, resulting from smog and air pollution [10]. The biodegradability of the natural cellulose from the plant fiber is considered the most vital and remarkable aspects of their utilization as filler in polymeric materials, due its abundance, low cost, low energy consumption, and non-toxicity [11,12]. In this regard, quite a number of works has now been undertaken, by numerous research groups, involving the development of natural cellulosic fibers. By-products of agricultural yields have been considered abundant, annually renewable, inexpensive, and sustainable sources for natural cellulose fibers. The by-products of main food crops, including wheat straw, corn husks, oat husks and leaves, tomato peels, garlic straw, sugarcane stalks, and soybean straw, have been studied as potential fiber sources [13,14,15,16,17]. By-products from wood, such as dust, sawdust, wood shavings, etc., can also be used as additives to bio-composite [18]. Besides plants, the cellulose can also be obtained by curtained types of bacteria, called bacterial cellulose (BC). Plant cellulose and BC could be turned to a cellulose solution using cellulose solvent, such as N-Methylmorpholine-N-oxide (NMMO). This solvent is an effective green solvent for industrial cellulose. The solution is supposed to be an entirely physical process, without any chemical change being caused in the pulp or solvent, and offer a better alternative, being faster, easier, and more reproducible than traditional technologies, as well as to transform the system from heterogeneous to homogenous [19]. Discovering alternative sources for natural cellulose in current use is crucial to have sufficient stock of cellulose fibers at affordable prices in the future. It is well known that chemical constituents of natural fibers significantly vary, due to their diverse origins and types [20]. Growing and harvesting conditions can also influence this variability, in terms of their physical and tensile properties in filament form [21,22,23]. Generally, the cellulose, hemicellulose, and lignin in a typical lignocellulosic fall within the range of 30 to 60%, 20 to 40%, and 15 to 25%, respectively. These lignocellulosic compositions greatly influenced the mechanical properties of the fibers and resulted in significant properties for the mechanical performance of the polymer composites [24].

It is common knowledge that rice (*Oryza sativa* L.) is a staple food for the people of Asia, especially Malaysia. Rice is the world’s second largest cereal crop, after wheat; however, it produces large amounts of harvest residues [25]. The residual rice wasted from the rice production process is rice husk (RH) and RS. The amount of RS production in Asia is about 90%, from 525 million tons per year, of paddy straw produced in the world [26]. This production is excessive to the needs of the industry, as only about 20% of RS has been used for purposes such as ethanol, paper, fertilizers, and fodders, and the remaining amount is either removed from the field, in situ burned, piled, spread in the field, incorporated in the soil, or used as mulch for the following crop [27]. 

The paddy plant consists of five important main parts, namely paddy, stalk, leaf, stem, and root. RS consists of all parts of paddy plants, except rice. RS contains 30 to 36% cellulose content, 19–32% hemicellulose, 5 to 18% lignin [28,29,30], and 5.5% silicon [31]. Cellulose, one of the main components of RS, is expected to play an important role in the near future, as a raw material for the production of bio-products and chemicals. High cellulose content in RS has encouraged researchers to divert its uses to a more pertinent utilization. Cellulose is a linear polymer, composed from aldehyde sugar, which is commonly known as D-anhydroglucopyranose units (C_6_H_10_O_5_). Individual cellulose chains are hydrophilic, due to large number of hydroxyl groups in the structure [20]. Native cellulose (cellulose I) is the most crystalline type, which is present in forms of Iα (triclinic unit) and Iβ (monoclinic unit) [32]. Cellulose is insoluble in water; the poor solubility is mainly attributed to the strong intramolecular and intermolecular hydrogen bonding between the individual chains [33]. Regardless of its poor solubility characteristics, cellulose has been employed in a wide range of applications, i.e., composites, coating, and food packaging, as well as blood purification membranes in the biomedical field [34]. 

The extraction processes have been performed by various procedures. Each method provides different benefits and drawbacks, related to the amount and quality of produced cellulose (composition and final properties). Thus, the aims of this current work are to produce cellulose from RS, via two different approaches, and characterize the acquired cellulose fibers. The first method is the pulping method that will minimize the damage of the cellulose, thus maintaining the pulp strength properties; however, this method will be difficult to achieve during the dissolving pulp process. The second method is the chemo-mechanical method. This treatment has less process time and drove to fibers with more homogenous diameter distribution. It will be the great alternative for the additive material in composites. The RS cellulose was to implement the characterization of the acquired cellulose. The RS cellulose was then transformed into nanocellulose via the dissolution process and surface modification treatment. This environmental nanocellulose can be used as an alternative nanofiller to the synthetic filler. The most common representatives of synthetic fillers are silicone, polymethylmethacrylate (PMMA), and polyacrylamide. In that way, agriculture waste can be turned into wealth, which also helps in managing the biomass effectively. These two routes were created with slightly altered parts, based on previous study procedures [35,36]. The selection of these two methods was in consideration of cost, safety, and time consumption during the process. The comparison is important, in order to know which method produced the better fibers for additives in composites. The chemical composition of RS cellulose samples was determined. The obtaining cellulose was characterized by FESEM, XRD test, and FTIR spectroscopy.

## 2. Experimental Details

### 2.1. Materials

Rice straw (RS), scientifically known as *Oryza sativa* L., is one type of lignocellulosic material. RS, used the present study, was obtained from a local plantation in Ulu Derdap Perak, Malaysia. The elements percentage of obtained RS is tabulated in Table 1. The RS was sorted, cleaned, and cut into small pieces, approximately 5 cm chips. The cut RS was oven-dried at 60 °C for 24 h, prior to the extraction process. Other reagents used were sodium hydroxide (NaOH, 2 to 18%), 1M hydrochloride acid (HCl), sodium chlorite (NaClO_2_, technical grade, 80%), acetic acid (CH_3_COOH), 4-methylmorpholine N-oxide (NNMO 97%, powder), 75% ethanol, and (3-Aminopropyl) triethoxysilane (APTES). All these chemicals were supplied by Merck (Merck KGaA, Darmstadt, Germany). The chemicals used were reagent grade and were used as received.

### 2.2. Extraction of RS Cellulose

**Figure 1:**
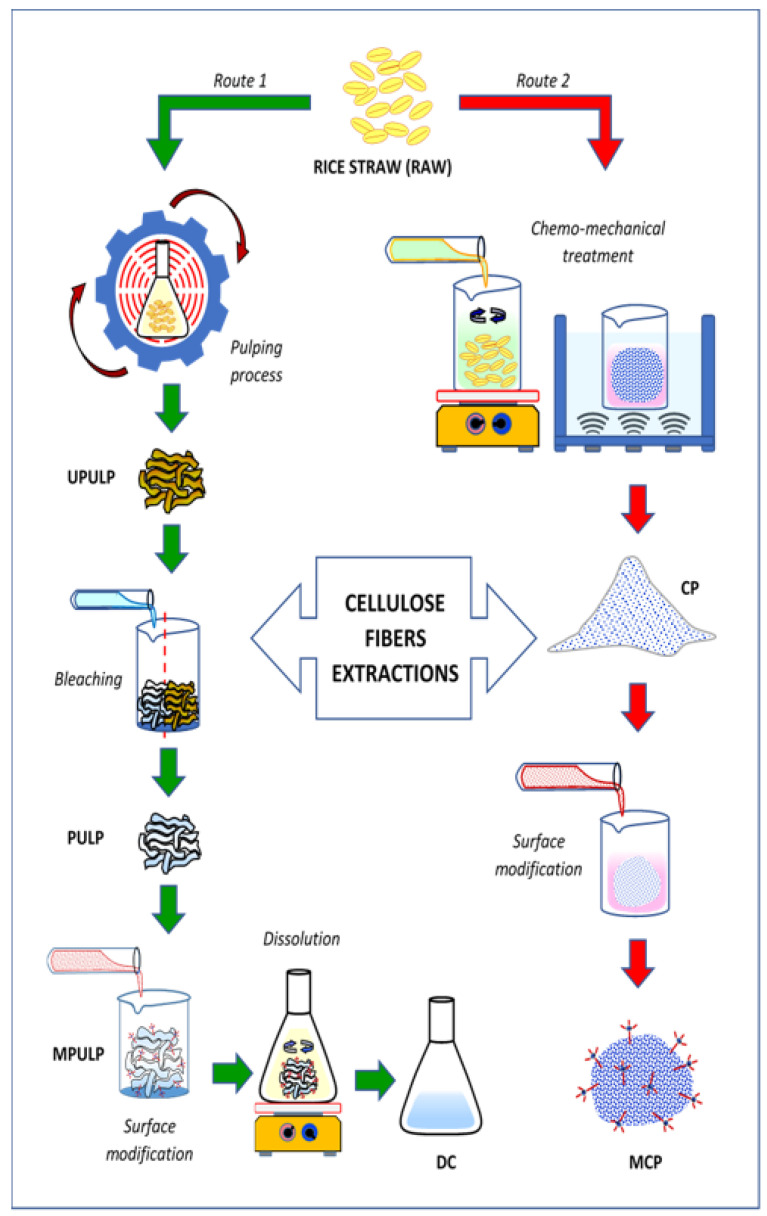
Schematic diagram of RS cellulose extraction methods.

Route 1: In this procedure, alkaline pulping process was used to isolate cellulose in RS from other constituents, such as hemicellulose, lignin, silica, and others. A total of 1 kg of RS was cooked in a rotary digester and stirred by rotating the reaction vessel under 8 kg/cm^2^ pressure at 170 °C. The RS was loaded into the digester, together with water and NaOH, and pulped according to the operating parameters for the 2-step pulping process, as specified in Table 2. For the first 3 h (pre-hydrolysis), the cooked pulp was washed using hot water and then continued to be cooked for another 3 h (soda pulping). After cooking, the fibrous product was separated from residual black liquor, through filtration using a fiber glass fabric. The filtered sample was disintegrated and washed repeatedly using distilled water. The fibers produced were named as unbleached pulp (UPULP). The wet pulp was then bleached in two sequences, i.e., (i) bleaching by NaClO_2_, CH_3_COOH, and distilled water for 120 min of treatment duration and (ii) bleaching by NaOH and treated 60 min. The pulps were washed using distilled water, until they reached a neutral pH. This 2-step bleaching process was repeated until the color of the cellulose fiber became off-white. Then, the cellulose was washed and dried under ambient conditions. The fibers produced was coded as pulp (PULP). The PULP went through the surface modification treatment, also known as the silanization process, before the dissolution process. This stage also used aminosilane as a coupling agent [37]. The method of cellulose surface modification referred to studies of Bendahou et al. [38] and Mohd et al. [39], with slight alteration. The modification was carried out by adding 1% *w/w* liquid of amino silane to a 5% *w/w* cellulose pulp in a mixture of 80/20 (*v/v*) water/ethanol solvent and was stirred for 2 h. The pH of the solution was adjusted to pH 4 by adding few drops of acetic acid and stirred continuously for 1 h. The pH was maintained at pH4. After 3 h, the heat was turned off and continuously stirred overnight. The PULP–aminosilane solution was centrifuged to remove the excess aminosilane that did not graft on the PULP; then, samples were freeze-dried for 24 h before being characterized under various analyses. This process was to cut off the time of dissolution process. After the silanization process, the soft, cotton-like fibers were observed. The silane modification process on the pulp fiber is essential because it sped up the production of the dissolve cellulose. After the process, the fibers become fluffier, and it was easier to dissolve during dissolution process. The fibers were named modification pulp (MPULP). After that, a mixture of NMMO solvent and distilled water was dissolved by stirring at 50 °C. The ratio of water added to NMMO was 1:4, respectively. The MPULP was then added into the mixture and stirred at 100 °C to dissolve the cellulose in the solvent homogeneously. The dissolving cellulose was named dissolve cellulose (DC). This study uses NMMO as a solvent because it is environmentally friendly and less expensive than other solvents, such as ionic liquid. An illustration diagram of RS cellulose extraction methods is showed in Figure 1.

Route 2: The extraction process involved a multistep procedure, included swelling, acid hydrolysis, alkaline treatment, bleaching, and ultra-sonication. For the swelling process, RS chips were soaked in of 17.5% NaOH solution for 2 h and washed using distilled water, producing RS pulps. The RS pulps were then dried at 30 °C for 24 h. The swollen pulps were then hydrolyzed, using 1M of HCl at 70 to 90 °C for 2 h at a constant stirring speed. The hydrolyzed pulps were washed using distilled water, until the neutral pH was attained, and oven-dried for 24 h. Then, the pulps were alkaline treated using 0.02 M of NaOH for 2 h at 70 °C to 90 °C, at a constant stirring speed, followed by washing and drying. The treated fibers were bleached with NaClO_2_ at 60 °C for 1 h to remove soluble lignin. Finally, the bleached cellulose fiber was sonicated in distilled water, using a water-bath ultrasonicator, for 1 h and dried at 50 °C for 24 h. The fiber produced was named cellulose powder (CP). Surface modification on CP was done using 1% of aminosilane. The CP was treated with aminosilane for 1 h at 70 °C at a constant stirring speed. Next, the fibers were soaked for 2 h before being washed and dried for 24 h at room temperature. The fibers were labeled as modified cellulose powder (MCP).

### 2.3. Chemical Analysis

Proximate chemical analysis was conducted on dry RS, PULP, and MPULP samples, according to the following standard methods:

- Extractives (ethanol/toluene solubility): TAPPI T 204 CM-97;
- Holocellulose: TAPPI T 203;
- α-cellulose: TAPPI T 203;
- Lignin: TAPPI T 222;
- Ash: TAPPI T 15.

### 2.4. Morphology Observations

Prior to the analysis, a thin layer of cellulose fiber was distributed on the sticky surface of a sample holder. The morphology of RS and RS cellulose was evaluated by FESEM model, ZEISS GeminiSEM 500 (Carl Zeiss, Oberkochen, Germany), using an Everhart–Thornley secondary electron (ET-SE2) or InLens mode detector with gold sputtering. The imaging conditions were a working distance of 2–4 mm, aperture size 30 μm (standard aperture), high vacuum mode, and imaged at accelerating voltage (0 V–30 kV).

### 2.5. XRD Test

XRD was performed to investigate the crystallinity and crystallite size of the produced RS cellulose samples by a Bruker instrument model, D8 Advance (Bruker, Billerica, MA, USA). The instrument used Cu-Kα radiation (λ = 0.15418 nm) at 40 kV and 40 mA. Scattered radiation was recorded in the angular range (2θ) of 2–40°. The sample configuration was a flat sample bracket with a specimen length of 10 mm. 

The crystallinity index (CI) and crystallite size of the RS cellulose samples was calculated from experimental diffraction pattern, using the XRD peak height method, developed by Segal and co-workers [40]. Segal’s method is an empirical method for estimating the degree of crystallinity of native cellulose using an X-ray diffractometer. This method examined the changes in XRD spectra during the decrystallization of cellulose by chemical and mechanical treatment. The purposed method was for experimental measurements to allow for the rapid comparison of cellulose samples. *CI* was calculated from the ratio of the height of the 002 peak (*I*_002_) and height of the minimum (*I_am_*), between the 002 and 110 peaks. Segal’s method is shown in Equation (1) [40]:

(1) $$CI=\frac{\left(I_{002}-I_{am}\right)}{I_{002}}\times 100$$

where *I*_002_ is the intensity of the crystalline peak, at the maximum 002 peak for I_β_ and cellulose II at 2θ, between 21° to 23°, and *I_am_* is the amorphous intensity between the 110 and the 200, at 2θ = 18° [32], while for cellulose II, the *I_am_* peak is at around 2θ = 16° [41].

The crystallite size, perpendicular to the 002-lattice plane. The average crystallite size was computed according to the Scherrer equation [42] (Equation (2)):

(2) $$D=\frac{0.89\lambda }{B\cos\theta }$$

where *D* is the size of crystallite, perpendicular to the plane, 0.89 corresponds to the values Scherrer constant (K), *λ* is the X-ray wavelength of the radiation (0.15418 nm), *B* is the full width half maximum (FWHM) in radian of the reflection of lattice planes, and *θ* and is the corresponding Bragg angle [43].

### 2.6. FTIR Spectroscopy

The vibration characteristics of chemical functional groups in the cellulose samples were detected using infrared spectroscopy. A small amount of cellulosic samples were placed and pressed the sample into ultra-thin pellets. FTIR spectra of cellulosic samples were recorded in the transmittance range of 400—4000 cm^−1^, by the resolution 4 cm^−1^, using a Spectrum 400 FTIR (Perkin Elmer, Waltham, MA, USA)

## 3. Results and Discussion

### 3.1. Physical Observations of Extraction of Cellulose from RS

In this study, two different routes, i.e., Routes 1 (pulping method) and 2 (chemo-mechanical method), performed the extraction of RS cellulose. By Route 1, the extracted cellulose unbleached pulp (UPULP) presented as yellowish lumps. After the alkaline treatment, insoluble lignin was still traced in the pulp, as evident in the yellowish color. Prior to the dissolution of the cellulose, the pulp was bleached in a mixture of solution containing NaClO_2_, CH_3_COOH, and water to remove non-cellulosic residues and followed by the surface modification treatment to the pulp (MPULP). The bleaching process was due to the rapid discolouration during storage of these materials, most of which have low initial brightness. Bleaching the RS and other non-woody fibrous raw materials has proved difficult [44]. Apparently, the cellulose formed in NMMO solvent in a homogenous form, and the dissolving cellulose (DC-Route 1) was retained in liquid form to preserve its structure.

**Figure 2:**
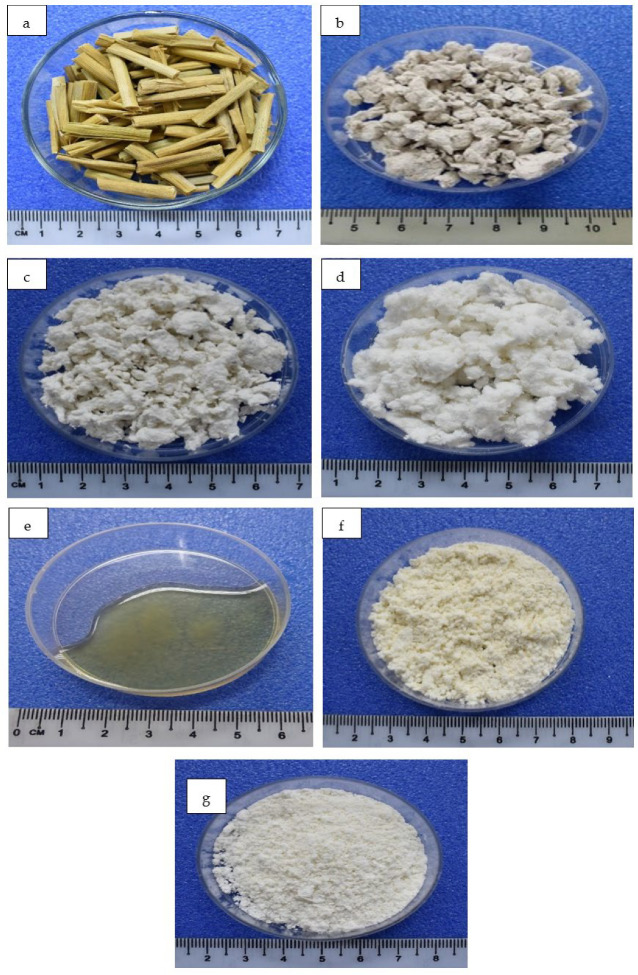
Photographs of (**a**) RS chip, (**b**) unbleached cellulose pulp (UPULP), (**c**) bleached cellulose pulp (PULP), (**d**) modified cellulose pulp (MPULP), (**e**) dissolve cellulose (DC), (**f**) cellulose powder (CP), and (**g**) modified cellulose powder (MCP).

The structures of extracted cellulose were evaluated, as shown in Figure 2. Visually, the raw RS was yellowish-brown in color. Via Route 2, the extracted cellulose powder (CP) was found to turn whitish-yellow, due to the residue of lignin content in the structure. After the bleaching step, almost all the remain lignin is removed. Therefore, the brightness of the CP was increased [36]. The dispersion of NaOH in the amorphous area disrupted the intermolecular bonds, which was due to the internal stress in plant cell wall, thus inducing the removal of the non-cellulosic parts of RS [45]. As the process was continued, the modified cellulose powder (MCP) was attained in a more whitish color. The MCP was obtained by the surface modifications process. After the process, it could be seen in the MCP sample that the surface was more a fine structure. This observation is due to the introduction of molecular chain onto the surface of cellulose powder [46].

The alkaline treatment was mainly carried out to remove the soluble lignin, residual hemicellulose, and pectin [47]. This scenario signified that the lignin and other non-cellulosic constituents in the raw RS were effectively dissolved and removed by the alkaline treatment, using 0.02 M NaOH, and the subsequent bleaching process, using NaClO_2_ solution, with evidence of the produced whitish cellulose fiber. One of the most important steps in chemo-mechanical treatment is the acid hydrolysis process. In this study, hydrochloric acid hydrolysis was used to enhance the cellulose fibers. A previous researcher [48] reported that the fibers carried out from hydrochloric hydrolysis exhibited a larger ratio than expected, compared to the sulfuric acid hydrolysis. Moreover, this will be better dispersion in the polymer matrix.

### 3.2. Chemical Composition

The result of the chemical composition percentage of RS, PULP, and MPULP are shown in Table 3. The chemical composition was done for the raw material and two cellulose samples produced by the pulping method, which are the bleached and modified pulp. Chemical composition analysis was conducted for an invaluable composition, component, and impurities in the materials, such as RS.

As it can be seen, the cellulose content increased from 53.02% to 84.9% via chemical treatment (pulping process until bleaching stage). The holocellulose is the total amount of cellulose and hemicellulose and is obtained by removing the extractives and the lignin from the original natural material. The hemicellulose contents were decreased from 22.77% to 12.50% (PULP) and 19.80% (MPULP) after chemical treatment. The hemicellulose with an amorphous structure, which has low molecular weight, was dissolved in alkali and acidity media. Thus, the major percentage of hemicellulose was removed from the fibers after the chemical purification. In addition, the lignin content was reduced from 30.98% to 1.03% (PULP) and 2.94% (MPULP). Details of aggregation of lignin to other components of lignocellulosic fibers have not been determined entirely yet, but this approach is dominant that lignin–hemicelluloses bonds are more probable than lignin–cellulose bonds. Thus, by removing the hemicelluloses, the structure of lignin is more accessible. Other workers demonstrated that, with alkali treatment before bleaching step, a significant percentage of soluble lignin content is removed.

### 3.3. Morphological Analysis of Cellulose Fiber

**Figure 3:**
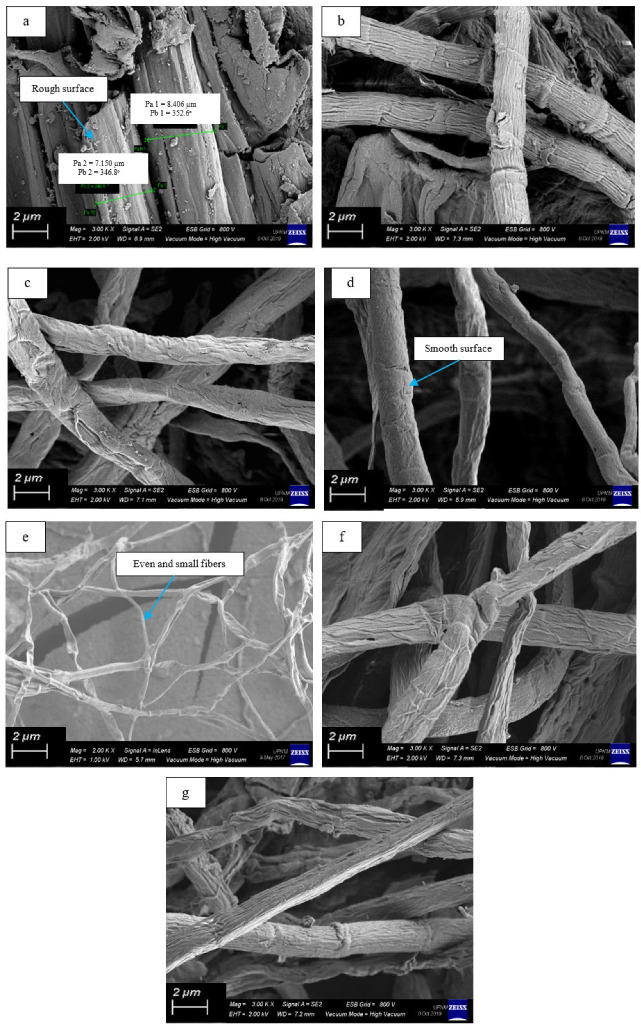
FESEM micrographs of (**a**) RS chip, (**b**) unbleached cellulose pulp (UPULP), (**c**) bleached cellulose pulp (PULP), (**d**) modified cellulose pulp (MPULP), (**e**) dissolve cellulose (DC), (**f**) cellulose powder (CP), and (**g**) modified cellulose powder (MCP).

To visualize the morphological changes in the extracted cellulose, FESEM observation was performed in all studied samples. Figure 3 show the fiber surface morphology at 2000× magnification. The fiber had fewer surface fines, due to the extraction technique used in this research. However, the surface shows that the fiber had a rough and irregular surface. After the surface modification and dissolving processes, the surface fibers became even and reduced in average diameter fibers. Table 4 shows the sample of raw RS, presented as a cluster of glued fibrillated fibers. An average diameter of the fiber bundles from RS was 7.78 µm. The surface fiber was considerably rough and irregular in shape that submerged with amorphous contents, including waxes, lignin, hemicellulose, cellulose, and some impurities. By extracting the cellulose, a significant change in the fiber was achieved. After the initial stage of extraction, micro-sized fibrillated fibers of UPULP, PULP, and MPULP were formed, with a large reduction of average diameter, i.e., of 4.04, 3.99, and 3.65 µm, respectively. The reduction of the average diameter was also observed for CP (3.83 µm), using the Route 2 method. This phenomenon signified that the cellulose micro/nano fibers from the RS waste material can be effectively extracted, according to both applied methods in this study. The surface of the cellulose fibers from R1 was rougher than R2, which suggests the higher removal of hemicellulose, waxes, and other impurities during the pulping and bleaching processes; these components provided rigidity, impermeability, and protection to the cellulosic biomass structure [50,51]. The dissolution of the MPULP in the organic NMMO solvent resulted in the fibers becoming flat and shortened, with an average diameter of 1.06 µm (DC). The organic solvent solution penetrated the amorphous regions of the cellulose during the cellulose regeneration and cleaved the β-1,4- linkage between the cellulose repeating units, thus breaking the outer layer of the fiber and forming a networking structure linked by H-bonding [9,52].

As the CP was subsequently treated via alkaline treatment, these fibers shriveled into an average diameter of 3.830 µm. The fiber surface became rougher and the outer layer of the fibers was disrupted and cracked at certain parts of the inner structure, revealing the fibril strand. The ordered crystalline arrangements appeared due to the formation of inter and intramolecular H-bonding between the hydroxyl groups. The H-bonding hinders the free movement of cellulosic chains, and they were bonded in a networking structure [53,54]. However, the reducing values of MCP and DC induced uniform size distribution, after both the modification and dissolution treatments.

### 3.4. Phase and Crystallinity of Cellulose

**Figure 4:**
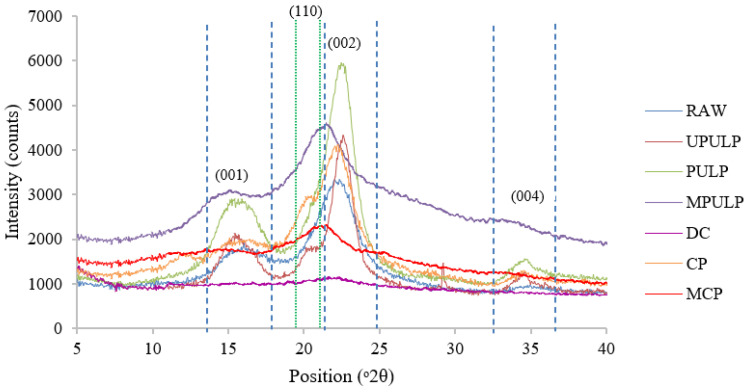
X-ray diffraction spectra of extracted cellulose fibers from RS.

Microfibrils are formed by self-assembly and multiple cellulose chains. They are composed of crystalline and amorphous regions [55,56]. To visualize structural changes, the studied samples were examined by X-ray diffraction (XRD) analysis. According to ICDD, diffraction peaks of native cellulose are located around 14.90° (001), 16.49° (110), and 22.84° (002) [57]. As shown in Figure 4, three characteristic peaks of raw RS at 14.90° (001), 16.20° (110), and 22.22° (002) were identified on the cellulose I lattice planes.

The analysis of crystallography, using XRD, was performed to confirm the polymorph of RS and the extracted cellulose fibers. At the initial stage of Route 1, the sharp peak was observed on the UPULP and PULP samples (at primary lattice plane 002), and the peak at 004 plane appeared after the chemical treatment in the pulping process. The UPULP peaks were at 2θ =15.7° (001), 20.50° (110), 22.6° (002), and 34.8° (004), and the PULP was at 2θ = 15.8° (001), 20.6° (110), 22.50° (002), and 34.7° (004). Meanwhile, the treatment of rice straw cellulose pulp in amino silane solution, which was produced MPULP sample and followed by dissolution process by NMMO solution (DC sample), led to a reduction of the crystallinity of cellulose I, which resulted in a change of the crystallinity after modification of cellulose surface. The peak of MPULP was at 2θ = 12.2° (001), 20.7° (110), and 21.7° (002), and the peak for DC was at 2θ = 15.6° (001), 20.6° (110), and 22.2° (002). Several researchers reported that, after the dissolution in mild acid, the regenerated phase was considerably non-crystalline or amorphous [43,58]. However, in this current finding, 5% crystallinity of DC was slightly retained. This phenomenon was due to the peeling away of thin layers from the original crystallites, due to exposure in monohydrate NMMO solvent, thus retaining some molecular ordering. By utilizing NMMO, the extracted cellulose was not derivatized, but was dissolved, in order to offer homogeneous polymer solution. After the solvent removal, these thin layers had the possibility to form a paracrystalline phase that was substantially diverse from the typical amorphous cellulose and closer in structure to cellulose II [59]. The diffractogram of DC shows one broad peak between 2θ = 20° to 25°. This peak corresponds to the 002 peak. It also has a tiny peak between 2θ = 19° to 20°, which is refers to the 011 plane. The planes observed in the DC diffractogram were similar to the previous study [60]. These X-ray traces are in accordance with those commercial regenerated fibers; therefore, rice straw modification fibers can be classified as type II cellulose fibers [61].

After the extraction process by Route 2, the diffractogram show the peaks of CP at 2θ = 16.1° (001), 20.2° (110), and 22.2° (002). The intensity of CP, after the swelling process in 17.5% NaOH, with a slight detection of peak at 22.2°, corresponded to the 002 crystallographic plane. This scenario signified that the usage of alkaline medium extensively increased the crystallinity of the extracted cellulose from RS. As commonly reported, a transformation of cellulose I to II does occur in alkali concentrations above 10% NaOH [62]. As the treatment process was extended, with modifications using aminosilane solution, the intensity of the primary peak of MCP was reduced. The obtained peaks of MCP were at 2θ = 15.4° (001), 19.9° (110), and 22.6° (002). This phenomenon explained that the biopolymer, in the form of an altered cellulose I phase, reduced the crystallinity of the modified cellulose [63]. The X-ray diffractogram indicates the crystalline structure of cellulose II. The peak intensity was wide and reduced significantly, by 22.6° (002), after the silanization. The size of crystallite and the crystallinity of cellulose are affected by chemical and mechanical treatments. Cellulose II samples indicated lower crystallinity after the acid hydrolysis, silanization, and dissolution processes. As many studies have reported, cellulose II is more easily hydrolyzed than cellulose I. It is important to know that crystallinity is a crucial factor in influencing the properties of fiber, particularly in terms of chemical, mechanical, and thermal properties, which will ultimately affect the properties of the composite made from it [64].

To determine how the distinct chemical and mechanical treatments affect crystallinity, crystallinity values were determined and compared between rice straw cellulose sample. Table 5 shows the summary of XRD peaks (001,110, and 002) for extracted cellulose from RS. The peaks were compared with the previous study [41]

The crystallinity index of cellulose was determined using the Segal technique (1959) [40]. The crystallinity result of rice straw cellulose samples was tabulated in Table 6. At the initial extraction process, the CI of the samples slightly increased from 58.50% to 82.80% for UPULP-R1 and 64.50% for CP-R2. This finding was confirmed by XRD analysis, i.e., the higher intensity at the primary 002 plane peaks of both UPULP-R1 and CP-R2, as compared to RAW RS. This phenomenon signified that the removal of cellulose from non-cellulosic materials and dissolution of the amorphous region was efficiently achieved. However, after the extraction process was prolonged with subsequent treatment, the CI was significantly reduced for both celluloses that were treated by different routes. The crystallinity index of bleached pulp (PULP-R1) is 78.50%, and the MPULP-R1 is 61.10%. For the PULP sample, the crystallinity of cellulose pulp decreased after the bleaching process, which indicated that sodium chlorite (NaClO_2_) and sodium hydroxide (NaOH) could partially disrupt the crystalline area. This means that strong alkali treatment would result in a slight decrease in crystallinity, which can be ascribed to strong alkali, not only removing the amorphous region of cellulose but also partially destroying the crystalline ones [65]. Besides, the CI values of MPULP also decreased. This was due to the introduction of a silane coupling agent into the polycrystalline domains. The results obtained in this study are in agreement with [66]. Meanwhile, the CI of DC that was regenerated using NMMO solvent was only 33.50%. The dissolution of the cellulose in NMMO demonstrated that the process led to the disruption of the crystalline regions and formation of altered crystalline domains. A considerably low CI was due to the transformation of the crystalline cellulose I to a para-crystalline structure, which resulted from the structure derivation of the organic solvent [43]. Besides, the crystallinity of the sample decreased because of high temperature used during the dissolution process. The average temperature in this process ranged from 90 to 130 °C. This is probably due to thermal agitation inflicted in the sample, as a result of temperature rise causing the reaction to proceed more aggressively [67].

The crystallinity values of CP and MCP sample by Route 2 were 64.50% and 50.10%, respectively. The CP sample was obtained after chemo-mechanical treatment and presented an increased crystallinity, with respect to RAW, as the chemo-mechanical treatment effectively eliminates amorphous cellulose from the fibers, leaving crystalline cellulose. The increase in the crystallinity index occurs during acid hydrolysis of cellulose. It seems that reaction is completed within 3 h, thereby keeping the native crystalline structure, crystallinity, and crystalline size intact. However, further increase in reaction time, add 3 h for the silanization process, resulted in decrease in crystallinity. This scenario was attributed to the disordered cellulose phase, which resulted from the modification treatment. The size of the aminosilane molecules is greater than that of the-OH groups, i.e., the distance between the polymer chains increased after modification. It is well known that the energy of intermolecular attraction reduces as the distance between the polymer chains increases. The other reason is [68] aminosilane molecules have NH groups that have less electronegativity than OH; hence, the hydrogen bonds tend to be weaker in MCP. This will imply different reinforcing effect if the fillers incorporate into nanocomposites.

The crystallite size of the samples endured different processing approaches, which are also given in Table 6. The crystallite size of the studied samples was calculated using Scherrer analysis. Results show the crystallite size of the rice straw cellulose sample become smaller after the chemical process, compared to the Raw samples, which are untreated samples. The crystallite size of the Raw was 9.78 nm, while the crystal sizes of the obtained samples, after the pulping process, were UPULP 7.42 nm, PULP 4.94 nm, MPULP 3.38 nm, and DC 3.70 nm. The decrease in crystallite size is due to the increase in reaction time, i.e., UPULP was obtained after 3 h pre-hydrolysis process; further, the procedure was continued for 3 h, followed by 2 h of bleaching process to gain PULP. The crystallite sizes of the samples via chemo-mechanical treatment (Route 2) were CP 4.87 nm and MCP 4.19 nm. According to the analysis, Route 1 offered a more significant refinement of extracted cellulose (DC) crystallite, as compared to the Route 2 (crystallite size of MCP), which was 3.70 nm and 4.19 nm, respectively. This scenario suggested that a more efficient disintegration of micro-sized cellulose fibers into nanofibers was achieved via the pulping method, followed by the dissolution process. This finding was in agreement with Duchemin and co-workers [69], who suggested that the existence of para-crystalline matrix is one of crucial reason for the mechanical property enhancement of polymeric composites.

### 3.5. Structural Arrangement by FTIR Analysis

**Figure 5:**
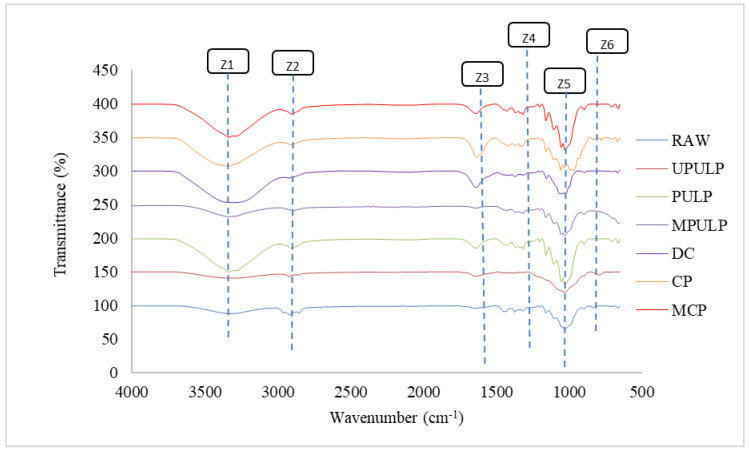
FTIR spectra of extracted cellulose fibers from RS. OH stretching (Z1), CH stretching (Z2), NH bending and vibration of NH_2_ (Z3). Si-CH_3_ (Z4), -Si-O-Si and -Si-O-C (Z5), NH wagging (Z6).

An alteration of the crystalline structure leads to a significant simplification of the spectra contour, through the reduction of intensity or even disappearance of the band’s characteristic of crystalline domains. A comparison in chemical and structural changes of components in the studied samples was performed via FTIR analysis. As shown in Figure 5, different routes of cellulose extraction from natural RS waste resulted in changes in the infrared band spectra. In this study, the typical bands for cellulose were observed for RAWs, such as OH stretching at 3327 cm^−1^, CH stretching at 2912 and 2847 cm^−1^, the C−O−C stretching vibration of the cellulose β-(1−4)-glucosidic linkage at 899 cm^−1^, and OH out-of-plane bending at 663 cm^−1^. The bonds mentioned above all appeared in the spectrum of all extracted cellulose samples, as well. The main vibrational peaks that were observed at 3600–3100 cm^−1^ were assigned to the intramolecular OH stretching at C-6 of cellulose. Increasing OH concentration suggested a reduction of hydrogen bonding in both treated samples. This scenario was a result of the removal of hydroxyl groups in reactions with corresponding solution during the cellulose treatments [70].

Additionally, as compared to the spectrum of the RAW, it was shown that some important changes in MPULP, MCP, and DC were observed after the modification of CNFs using aminosilane and NMMO solution. All spectra showed the emerging of small, new peaks, located at approximately 1600 cm^−1^ and 798 cm^−1^, which are attributed to NH_2_ bending and wagging, respectively. The same peaks that were reported by Abdelmouleh and co [71,72] are showing that the peaks are typical for the deformation modes of the NH_2_ groups of hydrogen, bonded to the OH functions of both silanol moieties and cellulosic substrates. The peak at 1623 cm^−1^ for MPULP and 1630 cm^−1^ for MCP, attributed to the NH bending and vibration of NH_2_ (Z3). Moreover, the intensity peak of Si-CH_3_ (Z4) at 1243 cm^−1^, as presented in the MPULP spectrum, was also observed in the MCP spectra, around 1280 cm^−1^, respectively. Bands for the -Si-O-Si and -Si-O-C (Z5) bonds at 1033 cm^−1^ and 1000 cm^−1^ for MPULP and 1056 cm^−1^ and 1000 cm^−1^ for MCP were overlapped with band C-O-C skeletal vibration, in the range 970–1250 cm^−1^. Z6 show the bands of NH wagging at around peak 792 for MPULP and MCP. These happened because of the functionalization process.

## 4. Conclusions

In conclusion, the chemical composition of cellulose fibers, after alkaline treatment, shows that the percentage of cellulose increased to 84.9% and slightly decreased after the surface modification process, about 75.9%. The morphological surface shows that the surface structure of RS cellulose samples is finer and smoother after the surface modification process, using silane for both methods. XRD analysis indicated that the reduction of crystallinity, after the silanization process, which was due to the chemical alteration induced by coupling agent solution, needs to be investigated extensively for efficient utilization. Although the crystallinity of CP and MCP decreased from 64.5% to 50.1%, they were retained in cellulose treated by Route 2. This finding can be reflected to the enhancement of mechanical properties during further usage, especially as a reinforcement in polymer matrices, which was attributed to the transformation crystalline cellulose I to paracrystalline structure.

RS isolated by the Routes 1 (pulping method) and 2 (chemo-mechanical treatment) processes can be a good resource for natural cellulosic products. The introduced methods can significantly be employed to defibrillate the cellulose bundles, where the results showed better accessibility of cellulose both modification and regeneration treatments. The applied methods resulted in the individualized RS microfibers and formation of network-structured cellulose fibers via the treatments. Chemo-mechanical treatment was found to be better, in terms of enhancing the physical properties of cellulose powder and modified cellulose powder, as well as reducing the fiber size. The homogenous diameter distribution of CP and MCP are most suitable candidate to be additive in fabrications of composites. This current finding provided an important outlook in producing cellulose nanofiber from abundant agricultural waste, which can be profitably utilized in a fabrication of new nanocomposites for various industries, including both high-scale products, such as packaging, automotive, precast concrete, and low scale products, such as cosmetic, aerogel, additive manufacturing, air, and water filtration.

## Figures and Tables

**Table 1:** The element content in RS.

Elements	Content (wt.%)
C	50.67
O	44.88
Mg	0.11
Si	1.99
P	0.80
K	1.20
Ca	0.36

**Table 2:** Operating parameters of RS pulping method.

Process	Reagent	Concentration (%)	Temperature (°C)	Time (h)
Pre-hydrolysis	NaOH	2	170	3
Soda pulping	NaOH	18	170	3

**Table 3:** Chemical composition of RS, PULP, and MPULP.

Chemical Component (%)	RS [49]	Bleached Cellulose Pulp (PULP)	Modified Cellulose Pulp (MPULP)
Extractives (ethanol/toluene solubility)	4.2	1.3	1.1
Holocellulose	75.79	97.40	95.70
Hemicellulose	22.77	12.50	19.80
α-cellulose	53.02	84.90	75.90
Lignin	30.98	1.03	2.94
Ash	12.00	0.27	0.26

**Table 4:** Average diameter and size distribution of cellulose fibers.

Sample	Diameter (µm)	Standard Deviation
RS, RAW	7.78	±11.67
UPULP	4.04	±6.69
PULP	3.99	±6.66
MPULP	3.65	±6.08
DC	1.06	±1.99
CP	3.83	±7.54
MCP	3.34	±5.11

**Table 5:** XRD peaks for extracted cellulose from RS.

Lattice Plane (*hkl*)	2θ (°) 001	2θ (°) 110	2θ (°) 002	Type of Cellulose
Range Standard value of cellulose I_β_	14.0–15.0	16.0–17.0	21.9–22.9	I_β_
Raw	14.9	16.2	22.2	I_β_
Standard value of cellulose II	12.1–16.0	19.8–21.0	21.0–22.9	II
UPULP	15.7	20.5	22.6	II
PULP	15.8	20.6	22.5	II
MPULP	12.2	20.7	21.7	II
DC	15.6	20.6	22.2	II
CP	16.1	20.2	22.3	II
MCP	15.4	19.9	21.6	II

**Table 6:** Crystallinity degree, crystallite size, and internal strain of the cellulose fibers.

Method	Sample	D-Spacing (nm)	Crystallinity Index (%)	Crystallite Size, D (nm)
	RAW RS	0.40	58.50	9.78
Route 1	UPULP	0.39	82.80	7.42
	PULP	0.40	78.50	4.94
	MPULP	0.42	61.10	3.83
	DC	0.40	33.50	3.70
Route 2	CP	0.30	64.50	4.87
	MCP	0.42	50.10	4.19

## Data Availability

Not applicable.
